# Exploring Gel-Point Identification in Epoxy Resin Using Rheology and Unsupervised Learning

**DOI:** 10.3390/gels9100828

**Published:** 2023-10-19

**Authors:** Eddie Gazo Hanna, Khaled Younes, Semaan Amine, Rabih Roufayel

**Affiliations:** College of Engineering and Technology, American University of the Middle East, Egaila 54200, Kuwait; khaled.younes@aum.edu.kw (K.Y.); semaan.amine@aum.edu.kw (S.A.); rabih.roufayel@aum.edu.kw (R.R.)

**Keywords:** epoxy, rheology, gel point, thermoset, principal component analysis (PCA)

## Abstract

Any thermoset resin’s processing properties and end-use performance are heavily influenced by the gel time. The complicated viscosity of resin as a function of temperature is investigated in this work, with a particular emphasis on identifying the gel point and comprehending polymerization. Rheology studies carried out using a plate-plate controlled stress rheometer under isothermal conditions were used to compare three experimental techniques for figuring out an epoxy resin’s gel point. We also look at the basic modifications that take place during polymerization. We verify the reliability of the three strategies by including Principal Component Analysis (PCA), an unsupervised machine learning methodology. PCA assists in uncovering hidden connections between these methods and various affecting factors. PCA serves a dual role in our study, confirming method validity and identifying patterns. It sheds light on the intricate relationships between experimental techniques and material properties. This concise study expands our understanding of resin behavior and provides insights that are essential for optimizing resin-based processes in a variety of industrial applications.

## 1. Introduction

Due to their exceptional mechanical, electrical, and thermal properties, as well as their one-of-a-kind combination of high strength and toughness, strong adhesion to a variety of substrates, and chemical resistance, epoxy resins have undergone extensive research and are frequently used in a variety of industries [1]. They also exhibit good dimensional stability and minimal shrinkage throughout the curing process [2]. Additionally, a suitable curing agent can be added, together with various fillers and reinforcements, to change the characteristics of epoxy resins [3].

When combined with different reinforcements like fiberglass, carbon fiber, or Kevlar, epoxy resins are frequently used as a matrix material in the field of composite materials to create polymer matrix composites (PMCs) with superior mechanical qualities [4,5]. Epoxy resins are used in the electronics industry to encapsulate and protect electronic components because of their electrical-insulating qualities [5]. Epoxy resins are employed as adhesives, varnishes, and sealants in the construction sector due to their high bonding ability and longevity [1].

The duration of time it takes in an epoxy resin system for the resin to transition from a liquid to a semi-solid state is referred to as the gel time [3]. This period is critical in setting the epoxy resin’s processing parameters and may affect the final attributes of the cured material [6]. While a shorter gel time may result in faster curing and less processing time, it may also limit the material’s ability to be worked [4]. A longer gel time allows for more processing and tooling time before the resin begins to cure. Many factors, including the type and amount of curing agent used, temperature, and the presence of accelerators or inhibitors, can influence the gel time of an epoxy resin system [7]. Increasing the temperature or using a more reactive curing agent, for example, can shorten the gel duration, whilst adding inhibitors can increase the gel period [1]. The type and amount of fillers or reinforcement elements used in the resin can also alter the gel time [2].

The gel point of epoxy resins has been determined using a variety of experimental methods [8]. Thermal Scanning Rheometry (TSR) has been shown to be an excellent approach for assessing gelation time [8,9,10,11]. The following rheological procedures for determining the gel point can be classified:

- Determination of the crossover point of the elastic modulus (G′) and viscous modulus (G″), or point where the loss tangent tan⁡δ=1 [12,13,14];
- Maximum of the tan⁡δ curve [15,16,17,18,19];
- The point at which tan⁡δ becomes independent of the frequency (*ω*) [20,21];
- The moment when the viscosity of the reacting system becomes infinite [22];
- The dependence of the relaxation modulus on a power-law relationship [23].

Additionally, methods based on dynamic mechanical analysis (DMA), where the viscoelastic nature of the material is a key property, are frequently used to measure gelation time. The maximum energy loss tangent or the modulus of elasticity increase are extrapolated to determine the gelation period [24,25].

Dielectric Analysis (DEA) measures the polarization of a resin in an alternating electric field, with its constraints being the sample’s restricted thickness and polar nature [8,26]. Differential Scanning Calorimetry (DSC) is another popular method for determining gelation time, in which two samples of resin are measured, fresh resin after mixing with the hardener and resin quenched immediately after gelation, and the reaction heats (ΔH) are compared to obtain the degree of conversion (α) versus time plot. The gelation time is the moment when the quenched sample of freshly prepared resin reaches φ [27,28,29]. Lastly, ultrasonic methods, which measure gelation time based on high-frequency (above 20,000 Hz) mechanical vibration damping, are also often described in the literature [30,31,32].

In recent years, Acebo et al. [33] used small-amplitude oscillatory shear (SAOS) methods to measure the gel time of epoxy/anhydride thermosets. Their results showed that G′, G″, and tan⁡δ values changed over time for a sample processed at 100 °C with a 5 Hz frequency. A notable change is observed between G′ and G″ at 30 min, indicating a shift from a liquid state to a more solid form. This change, denoting gel time, is frequency-dependent. It is more suitable to track the tan⁡δ  crossover, which can be deduced by comparing tan *δ* against time across various frequencies. Whenever graphs from different frequencies merge, it signifies a set gel time, regardless of the initial frequency. Utilizing this approach, Acebo et al. determined the gel times for multiple samples. They also pinpointed the vitrification or glass-transition temperature, which neared the reaction temperature.

In their research work about thermosets embedded with conductive nanoparticles [34,35,36], Chapartegui et al. focused on the rheological study of nanocomposites made by dispersing conductive carbon nanotubes, or CNT, within thermosetting epoxy resin bases. They measured the viscosity prior to curing to determine the amount of CNT required to form a continuous network and to assess the processability of the nanocomposite [34]. Within this framework, the creation conditions for multiwall carbon nanotubes (MWCNT)/benzoxazine buckypapers were explored [35]. Their work underscores the importance of rheology in buckypapers and provides insights into the optimal time and temperature for a resin to permeate a nanoparticle network. Using SAOS evaluations, they discerned that the inclusion of MWCNT expedited the curing time to reach the gel stage, though this acceleration diminished for higher concentrations surpassing the percolation threshold. Their comprehensive research, heavily grounded in rheological observations, culminated in the development of thermosets with exceptional electrical characteristics [35].

Recently, Chaloupka et al. [37] examined the simultaneous relationship between rheological and dielectrical measurements (DEA) on Hexcel RTM6, employing a unified system for both methods. A reusable in-mold sensor was utilized for dielectric evaluations, and calibration was conducted considering the cable’s response. When simultaneously analyzing dielectrical and rheological measurements during the curing process of the epoxy resin, it was observed that the identified values for glass-transition temperature, gel point, and viscosity aligned well. This congruence can be attributed to the fluctuation-dissipation theorem, which ties molecular dynamics to overarching mechanical properties of macromolecules. This study indicates that the metrics obtained from both DEA and rheology are driven by the same microscopic activities, particularly the dynamics linked to α-relaxation modes. As a result, both methods can be employed to detect similar phenomena, including glass transition and gel point.

In a research paper by Bekhta et al. [38], the viscosimetric and rheological behaviors of a new epoxy resin, Tetraglycidyl ether urea of bisphenol A (TGEBUA), and its composite were studied. The viscosity of the TGEBUA/Methanol system was determined using a VB-1423 Ubbelohd capillary viscometer. Furthermore, the study explored the rheological properties of the epoxy resin TGEBUA crosslinked with methylene dianiline (TGEBUA/MDA). Different composites of TGEBUA/MDA/TGEMDA + TSP were also formulated using two charges, tetraglycidyl ether of methylene dianiline (TGEMDA) and trisodium phosphate (TSP), at varying percentages (0%, 10%, and 15%). These properties were assessed using a RHM01-RD HAAKE rheometer. The study found that various formulated composites exhibited distinct rheological behaviors, largely influenced by the interaction between the epoxy resin and the fillers, which affected the mobility of the TGEBUA macromolecular chains.

HexPly^®^ M21 (Hexcel), an epoxy resin commonly used in aeronautics, was chosen for this study because it is a high-performance material with superior mechanical, thermal, and chemical resistance qualities that is employed in a variety of industrial applications [29]. It is a two-part epoxy system composed of a resin and a hardener that must be combined before use [30]. This resin cures to generate a robust, durable composite material that is resistant to moisture, chemicals, and extreme temperatures [31]. Its great mechanical strength is one of its key features, making it a perfect material for use in high-stress applications such as airplane structures and wind turbine blades [39,40].

This study aims to investigate how the complex viscosity of the resin varies with temperature. The best method for identifying the gel point will be attempted, and after that, we will try to comprehend the state of the matter and how polymerization works.

In addition, an unsupervised machine learning technique, the so-called Principal Component Analysis (PCA), will be applied to confirm or infirm the reliability of the three methods. Additionally, PCA will be used to look for patterns in order to reveal any hidden connections between the investigated methods from one side, and the different factors involved from another side.

## 2. Results and Discussion

### 2.1. Complex Viscosity

The complex viscosity, often denoted as $\eta^{*}$, is a measure used in rheology to describe the viscous and elastic behavior of complex fluids, such as polymer melts and solutions, when subjected to oscillatory shear deformation [41]. The loss modulus (G′) and storage modulus (G″) of a material can be used to describe its complex viscosity. The loss modulus represents the energy lost as heat as a result of viscous dissipation, whereas the storage modulus denotes the energy stored elastically. The complex viscosity-loss-storage moduli relationship is a measure of the material’s viscoelastic behavior [42,43].

**Figure 1:**
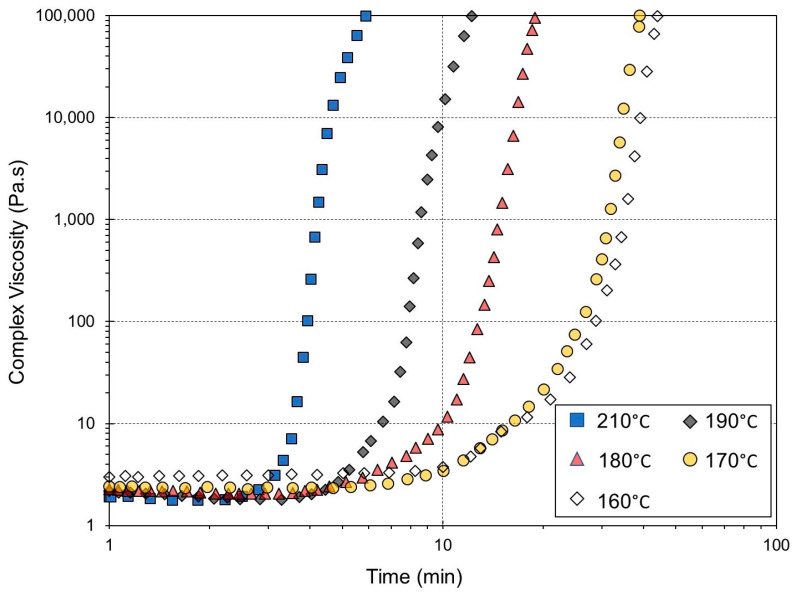
Effect of isothermal curing temperature on the complex viscosity.

Figure 1 illustrates the viscosity change in the epoxy resin during isothermal curing at a dynamic frequency of 1 rad/s. At each isothermal curing temperature, a steep rise in the complex viscosity value is observed, indicating a phase transition from liquid to solid. The rise in complex viscosity is guided by a Maxwell-like behavior [44]. The equation given below models the exponential progression of viscosity across varying temperatures:

(1) $${\eta^{*}(t)=\eta }_{0}^{*}+A_{0}\exp\left(\frac{t}{\tau_{n}}\right)\;,$$

where, $\eta^{*}(t)$ represents the complex viscosity, $A_{0}\;$ stands as a defining parameter, while $\tau_{n}\;$ signifies the relaxation of viscosity.

**Figure 2:**
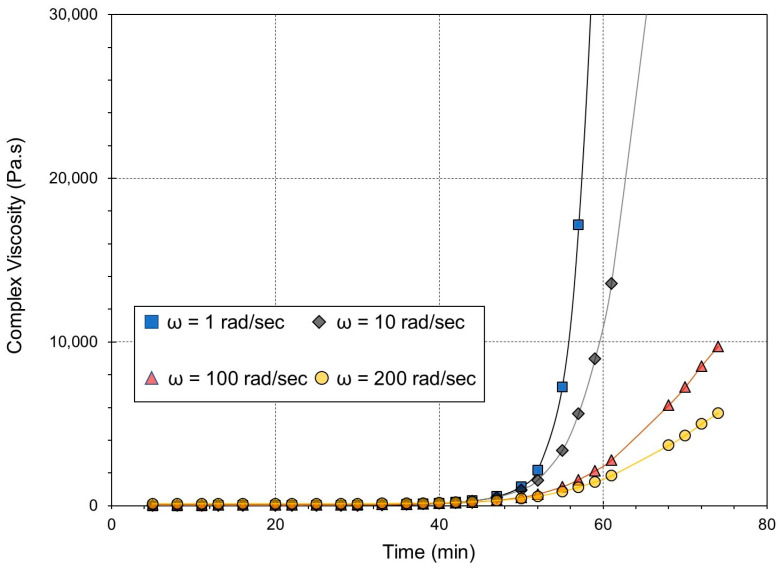
Effect of the frequency on the complex viscosity at 160 °C.

The effect of shear rate in the non-linear viscoelastic regime on the epoxy resin is shown in Figure 2. These results were obtained via an isothermal time-test rotation rheological experiment in which the shear rate was imposed on the sample by rotation of the moving plate of the rheometer. The isothermal temperature was set at 160 °C. As illustrated in this figure, during the initial stages of the reaction, the shear-viscosity curves at various constant shear rates exhibit minimal variation, indicating a lack of significant dependence on shear rate for the reactive mixture. This behavior can be attributed to the Newtonian characteristics of the low-molecular-weight precursors, which lack the capability to elongate under shear conditions. As the reaction progresses, the formation of molecular chains occurs. The application of shear rates induces the alignment of these molecular chains, resulting in a shear-thinning phenomenon and a dilution effect on viscosity caused by shear rate (referred to as shear-thinning behavior). Consequently, the viscosity of the reactive formulation at higher shear rates is lower than that observed at lower shear rates.

### 2.2. Gel Point

Several methods for calculating gel time have already been addressed. These methods do not always generate the same outcomes and must, therefore, be compared. Three methods are used in this section to establish the gel point of the epoxy resin. The first method involves determining the gel point by crossing the elastic modulus G′ and the viscous modulus G″. The second is based on the crossing of tan(*δ*) with time for various frequencies. The final one is based on the concept of infinite viscosity.

#### 2.2.1. Method 1: The CrossOver Point of the Elastic Modulus (G′) and Viscous Modulus (G″)

**Figure 3:**
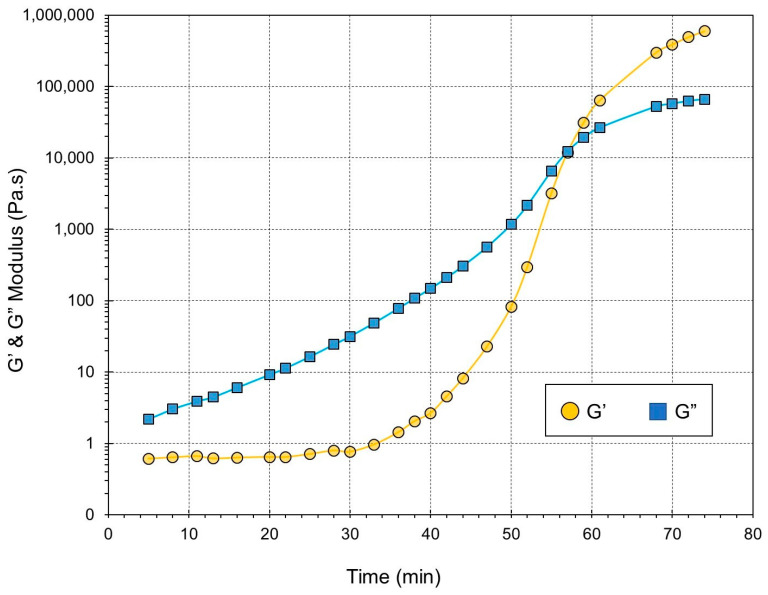
Loss modulus (G″) and storage modulus (G′) at 160 °C and *ω* = 1 rad/s.

Dynamic oscillatory measurements provide a reliable means of precisely determining the gel time of thermosetting systems. These experiments involve monitoring the changes in both the storage modulus (G′) and the loss modulus (G″) over time using small-amplitude oscillatory shear while maintaining a constant frequency. Figure 3 illustrates an example of the temporal evolution of G′ and G″ at 160 °C, demonstrating their relationship with respect to cross-linking time. The trends in storage and loss moduli changes at different isothermal temperatures are the same. Rheological properties like G′ and G″ are extremely sensitive to changes in molecular structure and phase transitions in thermosetting polymer systems [45,46]. Thermosetting polymer cross-linking can be modeled as a cluster-formation process [47]. Micro-gels are formed during the initial phase of the reaction with branched and partially cross-linked colloidal molecules [48]. The polymer continues to react, forming larger clusters of varying sizes that are distributed randomly throughout the system [49].

Rheologically, the thermosetting resin is in a liquid state during the early stages of curing, and viscous behavior dominates the first part of the curing process, followed by G″ > G′. Both dynamic moduli increase as the cross-link density and molecular weight of the curing polymer system increase. An infinitely large cluster extends throughout the system at the gel point, forming a three-dimensional continuous network and causing a crossover of the G′ and G″ curves [46].

The gel point has been defined as the crossover point of G′ and G″ during thermoset curing as a criterion for elasticity dominance in a reactive system [45,46,50,51].

**Figure 4:**
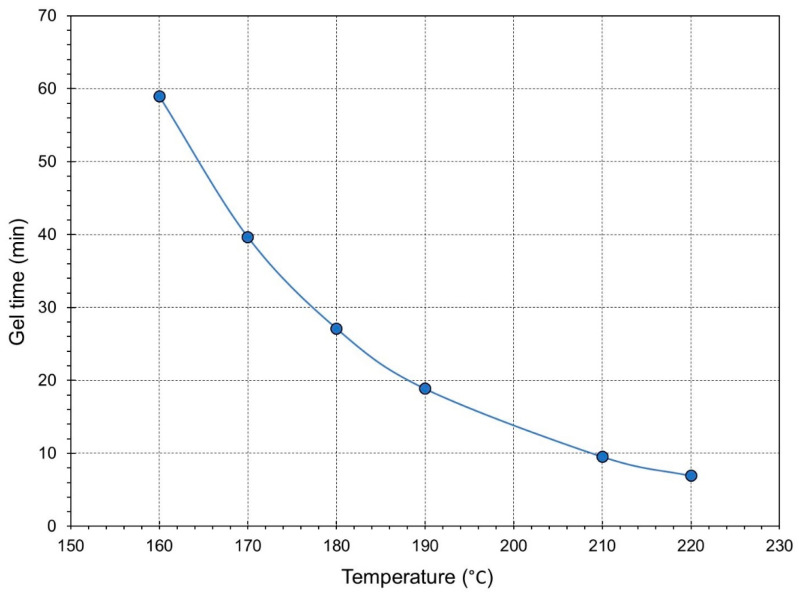
Effect of increasing temperature on the gel time.

Figure 4 depicts gel times obtained from the crossover points of G′ and G″ plotted against isothermal temperature. As shown in the graph, increasing the temperature of the measurement causes a decrease in gel time. This is due to higher reaction rates at higher temperatures. As expected, the gel time decreased as the rate of the crosslinking reaction increased with temperature.

Based on the dynamics near the critical point of gelation, predictions for the frequency dependence of the components of the complex modulus $G^{*}(\omega )=G{}^{\prime }(\omega )+i\;G{}^{\prime \prime }(\omega )$ have been made. At the gel point, the frequency dependence of the G′ and G″ can be represented by a power law over a wide angular frequency range: $G{}^{\prime }\sim G{}^{\prime \prime }\sim \omega^{∆}$ where, ∆=0.385, the loss factor ($\tan\delta =G{}^{\prime \prime }/G{}^{\prime }$) becomes frequency independent. The crossover of the loss-factor curves versus time at various frequencies can be used to determine the gel time [20,21].

#### 2.2.2. Method 2: The Point at Which tan (*δ*) Becomes Independent of the Frequency (ω)

**Figure 5:**
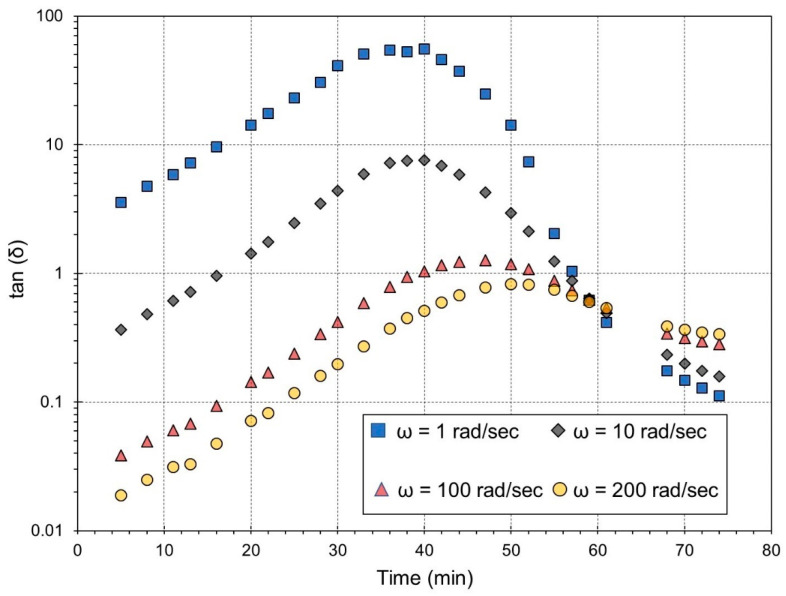
Curves of loss factor versus time at different frequencies of 1, 10, 100, and 200 rad/s and a temperature of 160 °C.

In this part, the loss factors intersection method, which is considered a multiple-frequency experiment, is used to validate the accuracy of gel-time calculations based on single-frequency experiments (the G′-G″ crossover method). Figure 5 shows the time evolution of the tan (δ) curves obtained at various frequencies at 160° C in a coincidence domain.

The loss factor starts to increase at the start of the reaction due to an increase in dynamic viscosity. After the initial scatter and loss-factor increase, tan⁡δ begins to decrease at a later reaction stage as the elastic part of the complex modulus (G′) operates due to the formation of elastically active cross-links before the gel point [46]. According to the results of the loss factors intersection method, gelation times at 160 °C are localized at 59 min.

#### 2.2.3. Method 3: The Moment When the Viscosity of the Reacting System Becomes Infinite

To determine the gel time, time-test oscillation measurements were performed at isothermal conditions in a temperature range from 160 °C to 220 °C. During the measurement, the viscosity initially increases slowly as the cross-linking reaction begins. However, as the reaction proceeds and the network formation accelerates, the viscosity increases more rapidly. Eventually, it reaches a point where it becomes practically infinite, indicating the gelation of the epoxy polymer.

The gel time is determined by analyzing the viscosity-time curve and it is identified as the moment when the viscosity abruptly increases and reaches a plateau or significantly deviates from the initial viscosity value [52]. Many studies have used the infinite-viscosity approach to analyze the gelation behavior of various thermoset resins such as epoxy, phenolic, and polyester resins, as well as to explore the influence of various curing conditions on the gel point. Table 1 shows the values produced using each approach for the various temperatures and at a shear rate of 1 s^−1^.

The corresponding gel time for every method follows a thermodependency according to an Arrhenius rule [53] and is given by the equation:

(2) $$t_{gel}=Ae^{\left(E_{a}/RT\right)}\;.$$

**Figure 6:**
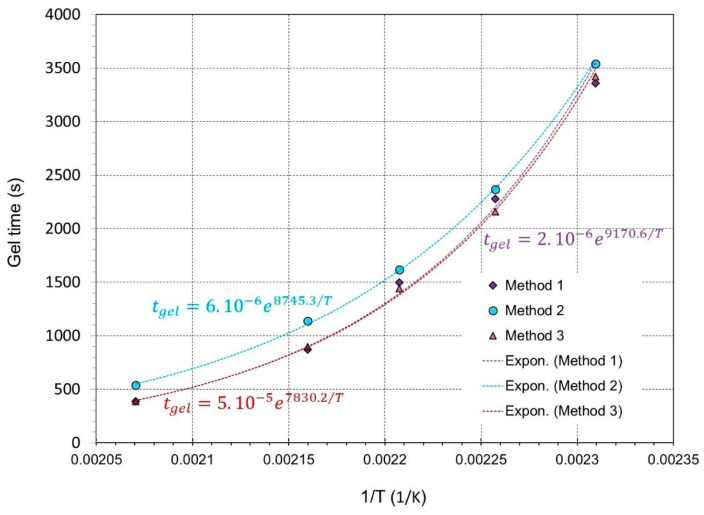
Gel time vs. temperature following the Arrhenius equation.

The three methods generate fairly comparable results in terms of duration, activation energy, and the pre-exponential constant of the Arrhenius law, as shown in Figure 6.

Based on the findings presented in Table 1, it can be deduced that there is no significant distinction between the time at which G′-G″ crossover occurs and the point of intersection of tan(*δ*) in relation to the overall time scale of gelation at each temperature. As a result, the validity of using the G′-G″ crossover as the gel-time reference point is confirmed, and the moment when the G′ and G″ curves intersect can be utilized as the gelation point for the epoxy formulation. In order to confirm or infirm the aforementioned findings, we attempted to apply PCA to the dataset of Table 1. Here, PCA is applied to seek a more sophisticated statistical comparision between the three methods, and probably to decipher any hidden patterns in the conventional bi-dimensional perspective.

### 2.3. PCA Results

**Figure 7:**
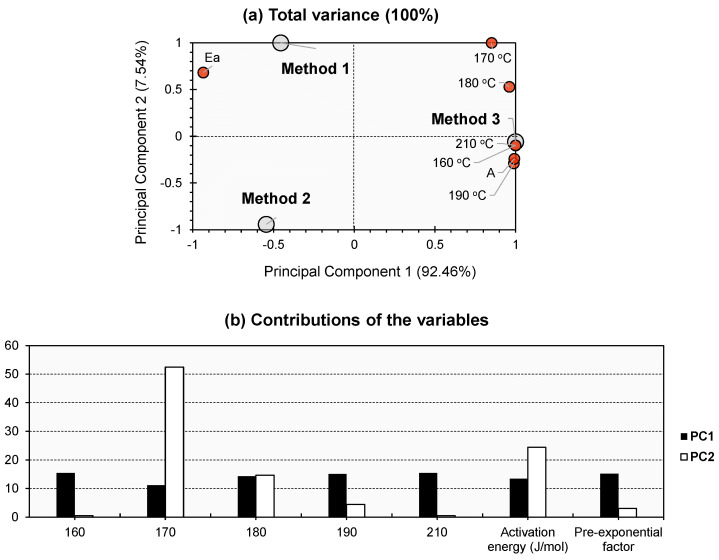
(**a**) PCA bi-plot for the dataset of Table 1. Gray bullets represent the individuals of the population (the three investigated methods). Red bullets represent the variables (different physical parameters). (**b**) % contribution of the investigated variables towards PC1 (black bars) and PC2 (white bars).

To establish the statistical validity of comparing the three explored methodologies, PCA was performed to examine the distribution of the data. Moreover, the application of PCA enhanced our comprehension of the dataset by revealing patterns that were previously obscured when examining the data solely from a two-dimensional perspective. Figure 7 illustrates the PCA bi-plot depicting the comparison of the three adopted methods for calculating the gel point. The initial two PCs demonstrated the cumulative variance, which accounted for 100% of the total variance (Figure 7a). This phenomenon can be attributed to the limited number of approaches employed within the examined population, as only three methods are engaged. The factors exhibited contributions ranging from 11% to 16% towards PC_1_, suggesting that they exerted comparable influences on the various approaches under investigation. A distinct pattern of dominance has been seen for the gel duration at a temperature of 170 °C in PC_2_ (52.466%; Figure 7b). The activation energy ($E_{a}$) made a significant impact, accounting for 24.432% of the overall result (Figure 7b). A modest result was obtained for the gel duration at a temperature of 180 °C, with a value of 14.649% (Figure 7b). It is worth noting that the temperatures mentioned above represent the range within which the investigated material underwent the curing process. This observation suggests that the curing range of the material has a significant impact on the gel-point approach, which exhibits substantial loadings (either positive or negative) along PC_2_. For the individuals, the three techniques exhibit significant dispersion along the PCA bi-plot, as depicted in Figure 7a. Method 1 had a moderate negative influence along PC_1_ and a high positive influence along PC_2_. In contrast, Method 2 demonstrated a moderate negative influence along PC_1_ and a high negative influence along PC_2_, positioned significantly opposite to Method 1. It is noteworthy that Method 1 exhibited a significant correlation with $E_{a}$, but Method 2 did not have a substantial beneficial impact on any of the factors. In accordance with the patterns observed in the variables, a majority of the latter had a substantial beneficial impact in relation to Method 3. The observed phenomenon of a high clustering of various variables using this method can be attributed to the significant representativeness of the first principal component (PC_1_) for the entire dataset. Therefore, it was observed that Method 3 exhibited a positive correlation with PC_1_, but PC_2_ had a minimal impact on the results.

Most of the varied patterns found in respect to individuals and variables can be explained using PC_1_ alone. However, along PC_2_, certain tendencies can also be seen. In light of the aforementioned assertion, the substantial loading of PC_2_, seen in both Method 1 and Method 2, would suggest the significance of the curing temperature in relation to this particular material, as well as its impact on $E_{a}$. The influence of Method 3 cannot be maintained. Hence, the latter is more likely to be influenced by the other elements. It is noteworthy that the pre-exponential factor has been tightly plotted along Method 3. From a kinetic perspective, factor A has been empirically identified as an indicator of molecular-collision frequencies. In contrast to $E_{a}$, the dependence of the rate constant on temperature is significant, as described by the Arrhenius law [54]. Based on the pronounced positive trend observed in the plot of Method 1, as well as the occurrence of a significant shift in temperature at 170 °C along PC_2_, it can be concluded that this approach is more sensitive to physical alterations in the polymer under investigation, which are influenced by variations in temperature. In contrast, the divergent trends observed in the plots of Method 2 suggest that this approach exhibits a limited sensitivity to the polymer’s physical changes induced by temperature.

## 3. Conclusions

The rheological behavior of epoxy resin was examined using a plate-plate controlled stress rheometer, revealing complex viscosity changes across a 1–200 rad/s frequency range. Three isothermal methods were compared to determine the resin’s gel point. The first highlighted the independence of the loss factor at the gel point from frequency, noting tan(δ) = 0.6. The second, a multi-frequency approach, validated gel times from single-frequency experiments, observing no significant time discrepancy for G′-G″ crossover. The third method identified when the reacting system’s viscosity became infinite, with results from all methods indicating a thermodependent gel time adhering to the Arrhenius rule.

PCA was used to confirm the consistency of these methods. Three main components captured variance tied to curing range and method sensitivity. Method 1 was the most sensitive to temperature changes, while Method 2 displayed limited reactivity. Method 3’s influence stemmed primarily from the first principal component.

Given these insights, future research incorporating numerical simulations could validate and bolster these experimental findings on epoxy resin behavior.

## 4. Materials and Methods

### 4.1. Sample Preparation

As previously stated, the epoxy matrix used in this study is commonly used in aeronautics. The HexPly^®^ M21 epoxy resin with hardener is provided on silicon paper. Before being studied, the resin is held at −18 °C (far below the glass-transition temperature T_g_ of −4 °C) to freeze the mobility of the macromolecular chains and avoid curing processes. Before beginning any studies, the resin is scraped from the paper, and clean samples measuring 0.55 g are prepared using a “Mettler Toledo” precision balance. These samples are then refrigerated until they are ready for use.

### 4.2. Rheological Experiments

#### 4.2.1. Time-Test Oscillation

Time-test oscillation measurements were performed at isothermal conditions in the temperature range from 160 °C to 220 °C. Frequency scans are performed using four selected frequencies (1, 10, 100, and 200 s^−1^), allowing for scans at varying speeds (25 s). To prevent excessive deformations and material loss, the fluid material is initially subjected to low stress. Since the original stress is no longer sufficient for deformation, the stress is increased when the material becomes rigid. The applied stress is adjusted for different levels of conversion. The preliminary stress is set to 10 Pa, and the optimal stress at the end of crosslinking is either 2000 Pa or 4000 Pa, depending on the temperature used. Three tests at each temperature are carried out to verify the results. A ramp is used to implement the stress change in stages. These tests are extremely sensitive to preparation quality, including weighing accuracy, speed, and efficiency.

#### 4.2.2. Time-Test Rotation

Measurements were carried out at shear rates of 1, 10, 100, and 200 s^−1^, which were imposed on the sample by rotation of the moving plate of the rheometer under isothermal conditions.

### 4.3. Dynamic Rheometer

The rheometer used in our tests is a TA instruments ARG2 Rheometer [55]. The ARG2 model is used to characterize complicated fluids. It has many capabilities, including for dynamic and steady-state testing, temperature control, and viscoelastic tests. The ARG2 is outfitted with cutting-edge software that offers a user-friendly interface and real-time data processing. It is composed of various components, such as a test station, a control computer, and an air drier. The test station houses the upper and lower plates, which are linked to the control computer and computer. The ARG2 software includes the ability to process all of the rheological quantities measured during the test, making it a comprehensive tool for fluid analysis.

The rheological measurements are carried out on a constant-stress-rate rheometer using a plan-plan arrangement using disposable plates of 25 mm diameter. After heating the oven to the specified test temperature, the clearance is adjusted accordingly. It is critical to ensure that no light is seen between the plates to ensure appropriate contact. The torque is then set by executing the appropriate command. This step is essential for conducting exact tests. The entire procedure takes around 15 min and must be completed every time the plates are changed. Following that, the spacing between the upper and lower plates is set to 1 cm. At this point, the oven is opened, and the pre-measured 30 g sample is placed between the plates, leaving an 8-micrometer space. The sample’s thickness is narrow enough to preclude any liquid flow caused by gravity. Since the material being introduced is in a liquid condition, the upper plate slides swiftly to the appropriate gap distance, avoiding any harm to the device. The precision of the measurements is largely dependent on the quality of the sample preparation and efficient sample placement.

### 4.4. PCA Overview

PCA is predominantly employed as a method for reducing the dimensionality of data in a wide range of disciplines, such as statistics, data analysis, and machine learning. PCA is a technique that enables the transformation of data with a high number of dimensions into a lower-dimensional space, while preserving a significant portion of the original data’s variability [56,57]. By employing data visualization techniques in this dimensionally reduced environment, one can acquire valuable insights regarding the inherent structure of the data, distinguish clusters, and identify probable outliers. The tool has been extensively utilized for data preprocessing, prior to implementing statistical validation techniques, and PCA may be employed to eliminate correlated features and mitigate multicollinearity. This contributes to enhancing the efficacy and comprehensibility of statistical models. In certain instances, PCA can be employed to ascertain the primary attributes within the dataset by assessing the extent to which each principle component (PC) contributes to the overall variance [56,57]. This process can help in the identification and selection of pertinent features for further investigations. The exact outcomes of certain statistical models rely on the fulfillment of key assumptions, including linearity, independence of variables, and constant variance, and PCA can be utilized to evaluate the linearity and independence of variables via the examination of a scatter plot of data points inside the PC space. Although PCA is not typically employed for model validation, the reduced-dimensional data derived from PCA can be utilized in statistical models to facilitate the validation process [56,58]. Assessing model performance, identifying overfitting, and evaluating assumptions made by the statistical model are potential benefits that can be derived using this approach. In certain instances, PCA has the potential to enhance the interpretability of statistical analyses by diminishing data complexity and emphasizing the most influential features. In other terms, it has the capacity to unveil concealed patterns that are rendered indiscernible using traditional statistical methodologies. PCA has numerous applications in mechanical analyses, enabling the extraction of useful insights, reduction of data dimensionality, and enhancement of the efficiency of diverse mechanical engineering processes. It has been commonly applied in mechanical analyses for many purposes such as vibration analysis [59], design optimization [58], process control and quality monitoring [60], and model reduction in finite element analysis (FEA) [57]. To the best of our current knowledge, there has been no previous attempt involving the application of PCA to statistically validate methods for determining the gel point.

### 4.5. PCA Adopted Methodology

PCA results were obtained using XLSTAT 2014 software, employing a methodology similar to that employed by Murshid et al. [61]. The technique under consideration is an unsupervised machine learning approach that relies on data-driven methods to reduce the size of a given dataset. The application of this reduction technique has resulted in improved visualization of a specific phenomenon. It allows for the exploration of hidden knowledge via the examination of correlations, both negative and positive. Additionally, it enables the assessment of the representativeness of the Principal Components (PCs) to the population under study. The  jth principal component matrix (Fi) is calculated by multiplying the unit-weighting vector ($U_{j}$) by the original data matrix M, which has dimensions m × n. The variables in this study are denoted as m, representing the number of variables, and n, representing the number of datasets. This notation is consistent with other studies [62,63,64,65]:

(3) $$Fi=U_{j}^{T}M=\sum_{i=0}U_{ji}M_{i}\;,$$

where U is the loading coefficient and M is the data vector of size n. The variance matrix M(Var(M)) is obtained by projecting M to U, should be maximized, and is as follows:

(4) $$Var\left(M\right)=\frac{1}{n}\left(UM\right){\left(UM\right)}^{T}=\frac{1}{n}{UMM}^{T}U\;,$$

(5) $$MaxVar\left(M\right)=Max\left(\left(\frac{1}{n}\right)\;{UMM}^{T}U\right)\;,$$

Since $\frac{1}{n}{MM}^{T}\;$ is the same as the covariance matrix of M(cov(M)), Var(M) can be expressed as:

(6) $$Var\;\left(M\right)=U^{T}cov\;\left(M\right)\;U\;,$$

The Lagrangian function can be defined by performing the Lagrange multiplier method as follows:

(7) $$L=U^{T}\;,$$

(8) $$L=U^{T}cov\left(M\right)U-\delta \left(U^{T}U-1\right),$$

For Equation (8), “$U^{T}U-1$” is considered to be equal to zero since the weighting vector is a unit vector. Hence, the maximum value of var(M) can be calculated by equating the derivative of the Lagrangian function (L) in respect to *U* as follows:

(9) $$\frac{dL}{dU}=0\;,$$

(10) $$cov\left(M\right)U-\delta U=\left(cov\left(M\right)-\delta I\right)U=0,$$

where

δ: eigenvalue of cov(M);
U: eigenvector of cov(M) (loading coefficient).

## Figures and Tables

**Table 1:** The gel times obtained at different temperatures according to the different methods—The parameters of the Arrhenius law of gel times.

	***ω***	**Temperature (** **°C** **)**							
	**1 s^−1^**	**160**	**170**	**180**	**190**	**210**			
		**Gel Time (min)**					**Activation Energy (J/mol)**	**Pre-Exponential Factor**	**Regression Coefficient**
Method 1		56	38	25	14.5	6.5	9170.6	2 × 10^−6^	0.9961
Method 2		56	36	24	15	6.5	8745.3	6 × 10^−6^	0.9965
Method 3		59	39.5	27	19	9	7830.2	5 × 10^−5^	0.9994

Method 1: Determination of the crossover point of the elastic modulus (G′) and viscous modulus (G″). Method 2: The point at which tan (*δ*) becomes independent of the frequency (*ω*). Method 3: The moment when the viscosity of the reacting system becomes infinite.

## Data Availability

Not applicable.

## References

[1] Handbook of Adhesive Technology. https://www.routledge.com/Handbook-of-Adhesive-Technology/Pizzi-Mittal/p/book/9780367572396.

[2] Epoxy Resins and Composites III (Advances in Polymer Science #78) (Paperback)|Barrett Bookstore. https://www.barrettbookstore.com/book/9783662151822.

[3] Pascault J.-P., Williams R.J.J. (2009). Epoxy Polymers: New Materials and Innovations.

[4] Introduction to Composite Materials Design. https://www.routledge.com/Introduction-to-Composite-Materials-Design/Barbero/p/book/9781138196803.

[5] Zhang G., Wang J., Xie Y., Shao Y., Ling Y., Chen Y., Zhang Y. (2023). CoS_2_ Particles Loaded on MOF-Derived Hollow Carbon Spheres with Enhanced Overall Water Splitting. Electrochim. Acta.

[6] Massingill J.L., Bauer R.S., Craver C.D., Carraher C.E. (2000). Epoxy Resins. Applied Polymer Science: 21st Century.

[7] Sukanto H., Raharjo W.W., Ariawan D., Triyono J., Kaavesina M. (2021). Epoxy Resins Thermosetting for Mechanical Engineering. Open Eng..

[8] Núñez-Regueira L., Gracia-Fernández C.A., Gómez-Barreiro S. (2005). Use of Rheology, Dielectric Analysis and Differential Scanning Calorimetry for Gel Time Determination of a Thermoset. Polymer.

[9] Mortimer S., Ryan A.J., Stanford J.L. (2001). Rheological Behavior and Gel-Point Determination for a Model Lewis Acid-Initiated Chain Growth Epoxy Resin. Macromolecules.

[10] Johnson L.M., Huffman N.D., Parameswaranpillai J., Hameed N., Pionteck J., Woo E.M. (2017). Rheology of Epoxy/Thermoplastic Blends. Handbook of Epoxy Blends.

[11] Laza J.M., Julian C.A., Larrauri E., Rodriguez M., Leon L.M. (1999). Thermal Scanning Rheometer Analysis of Curing Kinetic of an Epoxy Resin: 2. An Amine as Curing Agent. Polymer.

[12] Harsch M., Herzog F., Karger-Kocsis J. (2008). Cure-Induced Normal Force Development in Unfilled and Filled Epoxy Resins. J. Compos. Mater..

[13] Shah D.U., Schubel P.J. (2010). Evaluation of Cure Shrinkage Measurement Techniques for Thermosetting Resins. Polym. Test..

[14] Costa M.L., Botelho E.C., de Paiva J.M.F., Rezende M.C. (2005). Characterization of Cure of Carbon/Epoxy Prepreg Used in Aerospace Field. Mat. Res..

[15] Harran D., Laudouard A. (1985). Caractérisation de la gélification d’une résine thermodurcissable par méthode rhéologique. Rheol. Acta.

[16] Hayaty M., Beheshty M.H., Esfandeh M. (2011). A New Approach for Determination of Gel Time of a Glass/Epoxy Prepreg. J. Appl. Polym. Sci..

[17] Cadenato A., Salla J.M., Ramis X., Morancho J.M., Marroyo L.M., Martin J.L. (1997). Determination of Gel and Vitrification Times of Thermoset Curing Process by Means of TMA, DMTA and DSC Techniques. J. Therm. Anal..

[18] Ramis X., Cadenato A., Morancho J.M., Salla J.M. (2003). Curing of a Thermosetting Powder Coating by Means of DMTA, TMA and DSC. Polymer.

[19] Bilyeu B., Brostow W., Menard K.P. (2002). Separation of Gelation from Vitrification in Curing of a Fiber-Reinforced Epoxy Composite. Polym. Compos..

[20] Raghavan S.R., Chen L.A., McDowell C., Khan S.A., Hwang R., White S. (1996). Rheological Study of Crosslinking and Gelation in Chlorobutyl Elastomer Systems. Polymer.

[21] Lange J., Altmann N., Kelly C.T., Halley P.J. (2000). Understanding Vitrification during Cure of Epoxy Resins Using Dynamic Scanning Calorimetry and Rheological Techniques. Polymer.

[22] Hong B.T., Roh S.S., Kim D.S. (2004). Chemorheological Studies on a Dicyanate Resin Modified with Polyethersulfone. Polym. Int..

[23] Ng T., McKinley G. (2008). Power Law Gels at Finite Strains. J. Rheol..

[24] Beheshty M., Nasiri H., Vafayan M. (2005). Gel Time and Exotherm Behaviour Studies of an Unsaturated Polyester Resin Initiated and Promoted with Dual Systems. Iran. Polym. J..

[25] Stark W. (2013). Investigation of the Curing Behaviour of Carbon Fibre Epoxy Prepreg by Dynamic Mechanical Analysis DMA. Polym. Test..

[26] Thermal Analysis of Polymers: Fundamentals and Applications|Wiley. https://www.wiley.com/en-us/Thermal+Analysis+of+Polymers%3A+Fundamentals+and+Applications-p-9780471769170.

[27] Varley R.J., Hodgkin J.H., Hawthorne D.G., Simon G.P. (1996). Toughening of a Trifunctional Epoxy System. II. Thermal Characterization of Epoxy/Amine Cure. J. Appl. Polym. Sci..

[28] Gao J., Li L., Deng Y., Gao Z., Xu C., Zhang M. (1997). Study of Gelation Using Differential Scanning Calorimetry (DSC). J. Therm. Anal..

[29] Restrepo-Zapata N.C., Osswald T.A., Hernández-Ortiz J.P. (2014). Method for Time–Temperature–Transformation Diagrams Using DSC Data: Linseed Aliphatic Epoxy Resin. J. Appl. Polym. Sci..

[30] Acitelli M.A., Prime R.B., Sacher E. (1971). Kinetics of Epoxy Cure: (1) The System Bisphenol-A Diglycidyl Ether/m-Phenylene Diamine. Polymer.

[31] Lionetto F., Maffezzoli A. (2013). Monitoring the Cure State of Thermosetting Resins by Ultrasound. Materials.

[32] Zhang Y., Wang Y., Guo C., Wang Y. (2022). Molybdenum Carbide-Based Photocatalysts: Synthesis, Functionalization, and Applications. Langmuir.

[33] Acebo C., Picardi A., Fernández-Francos X., De la Flor S., Ramis X., Serra À. (2014). Effect of Hydroxyl Ended and End-Capped Multiarm Star Polymers on the Curing Process and Mechanical Characteristics of Epoxy/Anhydride Thermosets. Prog. Org. Coat..

[34] Chapartegui M., Markaide N., Florez S., Elizetxea C., Fernandez M., Santamaría A. (2010). Specific Rheological and Electrical Features of Carbon Nanotube Dispersions in an Epoxy Matrix. Compos. Sci. Technol..

[35] Chapartegui M., Barcena J., Irastorza X., Elizetxea C., Fernandez M., Santamaria A. (2012). Analysis of the Conditions to Manufacture a MWCNT Buckypaper/Benzoxazine Nanocomposite. Compos. Sci. Technol..

[36] Chapartegui M., Markaide N., Florez S., Elizetxea C., Fernandez M., Santamaria A. (2012). Curing of Epoxy/Carbon Nanotubes Physical Networks. Polym. Eng. Sci..

[37] Chaloupka A., Pflock T., Horny R., Rudolph N., Horn S. (2018). Dielectric and Rheological Study of the Molecular Dynamics during the Cure of an Epoxy Resin. J. Polym. Sci. Part B Polym. Phys..

[38] Bekhta A., Hsissou R., Berradi M., El Bouchti M., Elharfi A. (2019). Viscosimetric and Rheological Properties of Epoxy Resin TGEUBA and Their Composite (TGEUBA/MDA/TGEMDA + TSP). Results Eng..

[39] Alger M. (1996). Polymer Science Dictionary.

[40] Jones D.R.H., Ashby M.F. (2011). Engineering Materials 1: An Introduction to Properties, Applications and Design.

[41] Ramli H., Zainal N.F.A., Hess M., Chan C.H. (2022). Basic Principle and Good Practices of Rheology for Polymers for Teachers and Beginners. Chem. Teach. Int..

[42] Münstedt H. (2021). Rheological Measurements and Structural Analysis of Polymeric Materials. Polymers.

[43] Rahul R., Kitey R. (2016). Effect of Cross-Linking on Dynamic Mechanical and Fracture Behavior of Epoxy Variants. Compos. Part B Eng..

[44] Vijayan P.P., Puglia D., Kenny J., Thomas S. (2013). Effect of Organically Modified Nanoclay on the Miscibility, Rheology, Morphology and Physical Properties of Diglycidyl Ether of Bisphenol-A Epoxy/Carboxyl-Terminated (Butadiene-Co-Acrylonitrile) Blend. Soft Matter..

[45] Winter H.H. (1987). Can the Gel Point of a Cross-Linking Polymer Be Detected by the G′–G″ Crossover?. Polym. Eng. Sci..

[46] Cai J.J., Salovey R. (2001). Chemorheology of Model Filled Rubber Compounds during Curing. Polym. Eng. Sci..

[47] Muthukumar M. (1989). Screening Effect on Viscoelasticity near the Gel Point. Macromolecules.

[48] Malkin A.Y., Kulichikhin S.G. (1991). Rheokinetics of Curing. Polymer Compositions Stabilizers/Curing.

[49] Muzumdar S.V., James Lee L. (1996). Chemorheological Analysis of Unsaturated Polyester-Styrene Copolymerization. Polym. Eng. Sci..

[50] Núñez L., Gómez-Barreiro S., Gracia-Fernández C.A. (2005). Study of the Influence of Isomerism on the Curing Properties of the Epoxy System DGEBA(N = 0)/1,2-DCH by Rheology. Rheol. Acta.

[51] Dean D., Walker R., Theodore M., Hampton E., Nyairo E. (2005). Chemorheology and Properties of Epoxy/Layered Silicate Nanocomposites. Polymer.

[52] Dimier F. (2003). Reactive Systems Injection: Kinetic and Rheological Laws Determination and Modelisation. Ph.D. Thesis.

[53] 6.2.3.1: Arrhenius Equation. https://chem.libretexts.org/Bookshelves/Physical_and_Theoretical_Chemistry_Textbook_Maps/Supplemental_Modules_(Physical_and_Theoretical_Chemistry)/Kinetics/06%3A_Modeling_Reaction_Kinetics/6.02%3A_Temperature_Dependence_of_Reaction_Rates/6.2.03%3A_The_Arrhenius_Law/6.2.3.01%3A_Arrhenius_Equation.

[54] Atkins P., Atkins P.W., de Paula J. (2014). Atkins’ Physical Chemistry.

[55] Costello B. (2005). The AR-G2 Magnetic Bearing Rheometer.

[56] Jolliffe I. (2005). Principal Component Analysis. Encyclopedia of Statistics in Behavioral Science.

[57] Wang H., Ding X. (2013). Identifying Sources of Variation in Horizontal Stabilizer Assembly Using Finite Element Analysis and Principal Component Analysis. Assem. Autom..

[58] Jun T.A.O., Gang S.U.N., Liqiang G.U.O., Xinyu W. (2020). Application of a PCA-DBN-Based Surrogate Model to Robust Aerodynamic Design Optimization. Chin. J. Aeronaut..

[59] Su L., Shi T., Liu Z., Zhou H., Du L., Liao G. (2017). Nondestructive Diagnosis of Flip Chips Based on Vibration Analysis Using PCA-RBF. Mech. Syst. Signal Process..

[60] Harkat M.-F., Mourot G., Ragot J. (2006). An Improved PCA Scheme for Sensor FDI: Application to an Air Quality Monitoring Network. J. Process Control.

[61] Murshid N., Mouhtady O., Abu-Samha M., Obeid E., Kharboutly Y., Chaouk H., Halwani J., Younes K. (2022). Metal Oxide Hydrogel Composites for Remediation of Dye-Contaminated Wastewater: Principal Component Analysis. Gels.

[62] Younes K., Laduranty J., Descostes M., Grasset L. (2017). Molecular Biomarkers Study of an Ombrotrophic Peatland Impacted by an Anthropogenic Clay Deposit. Org. Geochem..

[63] Younes K., Grasset L. (2017). Analysis of Molecular Proxies of a Peat Core by Thermally Assisted Hydrolysis and Methylation-Gas Chromatography Combined with Multivariate Analysis. J. Anal. Appl. Pyrolysis.

[64] Korichi W., Ibrahimi M., Loqman S., Ouhdouch Y., Younes K., Lemée L. (2021). Assessment of Actinobacteria Use in the Elimination of Multidrug-Resistant Bacteria of Ibn Tofail Hospital Wastewater (Marrakesh, Morocco): A Chemometric Data Analysis Approach. Environ. Sci. Pollut. Res..

[65] Younes K., Grasset L. (2020). The Application of DFRC Method for the Analysis of Carbohydrates in a Peat Bog: Validation and Comparison with Conventional Chemical and Thermochemical Degradation Techniques. Chem. Geol..

